# Case report: enzyme replacement therapy for Fabry disease presenting with proteinuria and ventricular septal thickening

**DOI:** 10.1186/s12882-024-03499-w

**Published:** 2024-02-21

**Authors:** Zewei Chen, Bo Yin, Juan Jiao, Tianyang Ye

**Affiliations:** 1 Department of Nephrology, The First Navy Hospital of Southern Theater Command, Zhanjiang, Guangdong China; 2 Department of Internal Medicine, The First Navy Hospital of Southern Theater Command, Zhanjiang, Guangdong China

**Keywords:** Fabry disease, Enzyme replacement therapy, Agalsidase α, Fabry nephropathy, Genetic testing

## Abstract

Fabry disease (FD) is an uncommon, X-linked, lysosomal storage disease that causes defects in the glycosphingolipid metabolic pathway due to deficient or absent lysosomal α-galactosidase (α-Gal A) activity. This leads to the accumulation of globotriaosylceramide (GL-3) within lysosomes in a wide range of cells, including endothelial, cardiac, renal, and corneal cells, and consequently, the progressive appearance of clinical symptoms in target organs. Enzyme replacement therapy (ERT), which involves the exogenous supplementation of α-Gal A enzyme and has been successfully administered for treating FD.

Here, we report a case of a 37-year-old male with complaints of recurrent proteinuria and ventricular septal thickening. A renal biopsy revealed vacuolization and foamy changes in podocytes, and the presence of myelin-like bodies and zebra bodies. The white blood cell α-Gal A activity was very low, while the Lyso-GL-3 level was high. Additionally, genetic analysis revealed a gene variant c.902G > A p. Arg301Gln. The patient was diagnosed with FD, and subsequently received intravenous ERT with a dose of Agalsidase α (0.2 mg/kg, 17.5 mg every 2 weeks). Currently, the values of proteinuria and ventricular septum thickness remain stable during the 6-month follow-up. Initiating ERT at an early age can effectively decrease the deposition of GL-3, attenuate the progressive clinical manifestations of FD, and provide greater long-term benefits.

**Supplementary Information:**

The online version contains supplementary material available at 10.1186/s12882-024-03499-w.

## Introduction

Fabry disease (FD) is a multisystem lysosomal storage disorder caused by a mutation in the alpha-galactosidase (α-Gal A) gene located on the X chromosome [1]. This results in the accumulation of globotriaosylceramide (GL-3) within lysosomes in a wide variety of cells, including endothelial, renal, cardiac, and corneal cells [2]. The global incidence of FD is currently estimated to range from 1/22,000 to 1/3000, however, it may be significantly underestimated due to the diverse range of clinical phenotypes [3]. A Fabry Registry have revealed a long delay between the onset of initial symptoms and a diagnosis [4, 5].

The clinical presentation of FD demonstrates a broad phenotypic variability ranging from multiorgan involvement, called classic type, to pauci-symptomatic or non-classic forms, known as both late-onset phenotype [6]. The classic symptoms of FD typically initiate at an early age (childhood or adolescence) and are characterized by neuropathic pain, digestive manifestations, angiokeratomas, and exercise intolerance, progressing later in adulthood to cardiomyopathy, chronic renal disease, and cerebrovascular disease. However, late-onset forms can manifest in adulthood and commonly involve only the kidney and heart injury [7].

As FD is a progressive disorder, early diagnosis and timely initiation of treatment are important aspects of its strategies. Renal, cardiac, and neurovascular involvements are the main life-threatening complications that require initiation of enzyme replacement therapy (ERT) as a critical portion of comprehensive management to prevent disease-related complications [8]. ERT could significantly reduce the body GL-3 and Lyso-CL-3 deposition through exogenous α-Gal A supplementation, benefit in enhancing renal pathology and cardiac function, and mitigate the severity of neuropathic pain [9]. Commencing ERT at an early stage may be more efficacious than patients with more advanced stages of the disease.

Here, we report a case of a 37-year-old male who was diagnosed with FD with complaints of proteinuria and ventricular septal thickening, and subsequently received intravenous ERT with a dose of Agalsidase α (0.2 mg/kg, 17.5 mg every 2 weeks). FD is a progressive disorder, so early initiation of ERT has the potential to eliminate major organ damage, and yield enhanced long-term benefits.

## Case presentation

A 37-year-old male was admitted to our department with complaints of recurrent proteinuria and ventricular septal thickening. He denied having a history of fever, shortness of breath, chest distress, abdominal pain, diarrhea, joint pain, skin rash, or typical findings associated with FD, such as angiokeratoma, acroparesthesia, corneal opacities, and hyperhidrosis. Previous medical history of the patient showed a history of hashimoto thyroiditis and preexcitation syndrome. There was no evidence of heart disease, typical kidney disease, or FD in his family members. After admission, his body temperature, heart rate, blood pressure, height, and weight were 36.7 ℃, 72 bpm, 118/65 mmHg, 170 cm, and 87.5 kg, respectively. Physical examination showed no significant abnormalities, including hearing impairment, cornea verticillate, rash, and edema. Urinalysis revealed 1 + protein as well as positive red blood cell (RBC) count of 32/ul (the normal level is < 25/ul), and 24-h urine protein was 370.8 mg (the normal level is < 0.15 g/24 h). Laboratory tests showed normal blood biochemical levels, with a serum creatinine (Scr) level of 90.6 µmol/L, blood urea nitrogen of 4.16 mmol/L. The other laboratory tests were normal or seronegative, including blood routine test, coagulation function test, D-dimer determination, vitamin B1 and B12, folic acid, antithyroid peroxidase, immunoglobulin, autoantibodies, complements, and tumor markers. Ultrasonography showed normal-sized kidneys, while cardiac echocardiography revealed a thickened interventricular septum (14.9 mm; the normal range is < 13 mm).

**Fig. 1:**
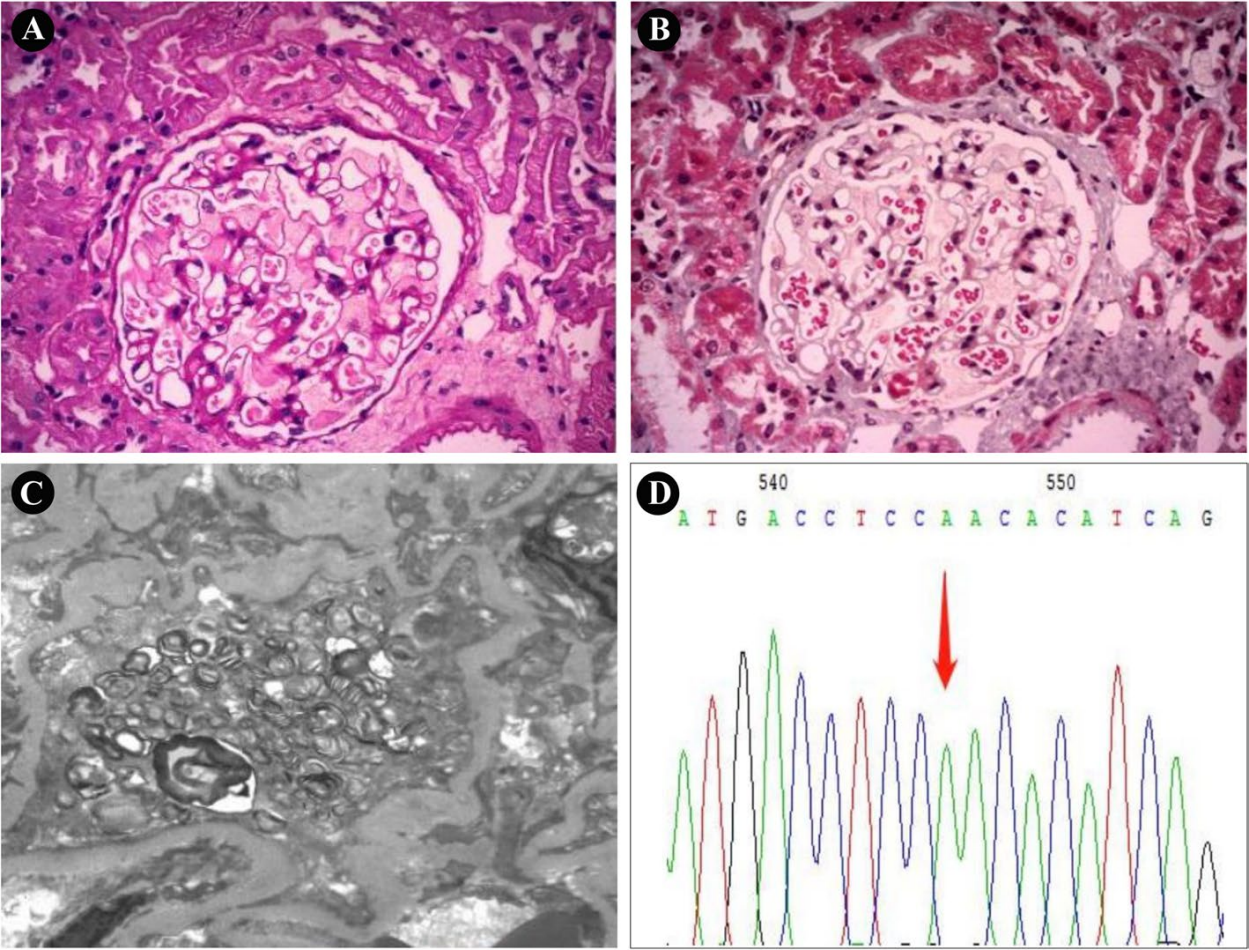
Results of renal pathological findings and genetic testing.** A** Light microscopy showing vacuolization and foamy changes in podocytes (PAS staining, × 400). **B** Light microscopy showing vacuolization and foamy changes in podocytes (Masson staining, × 400). **C** Electron microscopy showing epithelial cell swelling, spread foot process fusion, foamy changes with vacuolation, and myelin-like bodies and zebra bodies (× 200). **D** Genetic test showing variant c.902G > A p.(Arg301Gln)

Since no exact cause could be found, an ultrasonography-guided percutaneous kidney biopsy was performed. Light microscopy showed 31 glomeruli without any evidence of all-nodular sclerosis, segmental sclerosis and crescent. Vacuolization and foamy changes were observed in podocytes. In addition, the glomerular mesangial cells and matrix were slight proliferation (Fig. 1A and B). Immunofluorescence did not show any specific deposition of immunoglobulin or complement factors. Electron microscopy revealed epithelial cell swelling, spread foot process fusion, foamy changes with vacuolation, increased secondary lysosomes and presence of myelin-like bodies and zebra bodies. These above findings were consistent with FD nephropathy (Fig. 1C). The further tests showed that the white blood cell α-Gal A activity (0.40 µmol/L/h; the normal range was 2.4–17.65 µmol/L/h) was very low, while the Lyso-GL-3 level (14.71 ng/mL; the normal range was < 1.11 ng/mL) was high. In addition, we conducted a gene test, and variant c.902G > A p. (Arg301Gln) was detected, which is well recognized as a late-onset variant of FD (Fig. 1D).

The patient was finally diagnosed with FD. The patient initially underwent adjunctive treatment with an angiotensin receptor blocker (ARB; Losartan Potassium tablets, 100 mg/d) to control proteinuria. In May 2023, the patient received intravenous ERT with a dose of Agalsidase alfa (0.2 mg/kg, 17.5 mg every 2 weeks). No adverse events related to ERT were observed during the follow-up period, such as infusion-associated reactions, headaches, nausea, and rash. Throughout the 6-month follow-up, the patient remains in good health with no clinical symptoms. And the values of proteinuria and ventricular septum thickness remain stable.

## Discussion

Herein, we present a case of a 37-year-old male complaining of proteinuria and ventricular septal thickening, and then received intravenous ERT with a dose of Agalsidase α. To our knowledge, FD exhibits a broad spectrum of phenotypic variability ranging from multiorgan involvement to diverse individual differences. Patients diagnosed with FD experience a multisystemic disorder characterized by life-threatening complications, such as progressive renal insufficiency, cardiomyopathy, recurrent strokes, and neuropathic pain, which can lead to Fabry crises [10].

As FD is a progressive condition, it is imperative to confirm a definitive diagnosis to have timely access to favorite monitoring, appropriate management, and supportive treatment. The diagnostic method involves a comprehensive medical record, including family history, clinical and biochemical data, various imaging procedures, gene detection, and expert opinions [11]. The plasma α-Gal A activity below 1% strongly indicates a diagnosis of classic FD, and Lyso-GL-3 also holds clinical significance and has emerged as a robust biomarker for FD [12]. Furthermore, for patients suspected of cardiac disorder in FD should receive cardiac magnetic resonance imaging echocardiography, and a 24-h Holter electrocardiograph as early as possible to evaluate cardiac structure and function [13, 14]. For patients suspected to have renal involvement in FD, baseline assessment of renal function, estimated glomerular filtration rate (eGFR), urine protein quantitation, and renal imaging are suggested to carry out. Renal biopsy provides crucial information that may not be obtained from routine evaluation of kidney function and urinalysis, emphasizing the importance of renal biopsy in the initial assessment of all FD [15]. In our case, typical pathological presentations in electron microscopy present spread foot process fusion, foamy changes with vacuolation, and the presence of myelin-like bodies and zebra bodies. Genetic confirmatory testing is mandatory for all patients with FD, as it can identify the specific gene mutation, determine the clinical phenotype, and guide family screening [11, 16].

Since 2001, the treatment of FD has been revolutionized by the application of ERT, which can be used to facilitate the partial removal of the GL-3 deposition [12]. The following therapeutic effects can be achieved with ERT: stabilization of the myocardial thickness and reduction of left ventricular hypertrophy, stabilization of renal function or delay in the progression to end-stage renal failure, and alleviation of gastrointestinal complaints and improvement of neuropathic pain [17, 18]. ERT could stabilize the left ventricle (LV) mass or wall thickness, as supported by the Fabry Outcome Survey which showed no significant changes were observed in LV mass up to 5 years after initiation of ERT [19]. In a 6-month randomized controlled trial involving 26 patients with FD, the evaluation of creatinine clearance revealed maintained stable kidney function with periodically Agalsidase α therapy, while placebo treatment resulted in an 18% decline [20]. In our case, the patient received ERT shortly after his initial diagnosis, and both his proteinuria and ventricular septal thickness remained stable during the 6-month follow-up. In numerous reports on the therapeutic effects of ERT for FD, patients’ kidney function remained stable during follow-up after early initiation of ERT; however, these treatments demonstrated lower efficacy in patients at advanced disease stages or those with severe complications [21, 22]. This phenomenon may be explained by the fact that enzyme treatment is incapable of reversing the clinical courses. Despite the success of ERT, its clinical benefits are limited by the short half-life period of the recombinant enzyme, the incapacity to cross the blood–brain barrier, and the production of anti-drug antibodies [23]. Recently, advanced therapeutic approaches for the management of FD have been extensively investigated, both preclinically and clinically, including next-generation ERTs, gene therapy, and chaperone therapy [24]. These advanced therapeutic strategies will provide new opportunities to overcome the limitations of ERT.

In conclusion, we report a case of a 37-year-old male who was admitted to our department with complaints of proteinuria and ventricular septal thickening. Subsequently, he received intravenous ERT with a dose of Agalsidase α. As FD is a progressive disorder, initiating ERT at an early age can effectively decrease the deposition of GL-3, attenuate the progressive clinical manifestations of FD, and provide greater long-term benefits.

### Supplementary Information

**Supplementary material 1.**

## Data Availability

All data generated or analysed during this study are included in this published article [and its supplementary information files].
