# Supplementary material for: Case report: enzyme replacement therapy for Fabry disease presenting with proteinuria and ventricular septal thickening
Source: BMC Nephrol. 2024 Feb 21;25:61. doi: 10.1186/s12882-024-03499-w (PMC10882756; doi:10.1186/s12882-024-03499-w)
Supplement: Supplementary file 1 — Supplementary material 1. [file 12882_2024_3499_MOESM1_ESM.docx]

Supplemental Table 1. Baseline characteristics of the patient.

| Variables | Results |
| --- | --- |
| Age (year) | 37 |
| gender | M |
| Hemoglobin (g/L) | 147 |
| Albumin (g/L) | 48 |
| Creatinine (µmol/L) | 90.6 |
| Uric acid (µmol/L) | 318 |
| Total cholesterol (mmol/L) | 4.17 |
| Triglyceride (mmol/L) | 1.50 |
| IgG (g/L) | 8.03 |
| IgA (g/L) | 1.50 |
| IgM (g/L) | 0.81 |
| C3 (g/L) | 0.84 |
| HBV | N |
| HCV | N |
| Autoantibodies | N |
| 24 urinary protein excretion (mg/24h) | 370.8 |
| Uria red blood cell count (/ul) | 32 |
| Interventricular septum thickness (mm) | 14.9 |
| α-Gal A activity (µmol/L/h) | 0.40 |
| Lyso-GL-3 level (ng/mL) | 14.71 |

Abbreviations: M, male; Ig, immunoglobulin; N, negative; HBV, hepatitis B virus; HCV, hepatitis C virus; α-Gal A: α-galactosidase; Lyso-GL-3: lysosomal globotriaosylceramide.


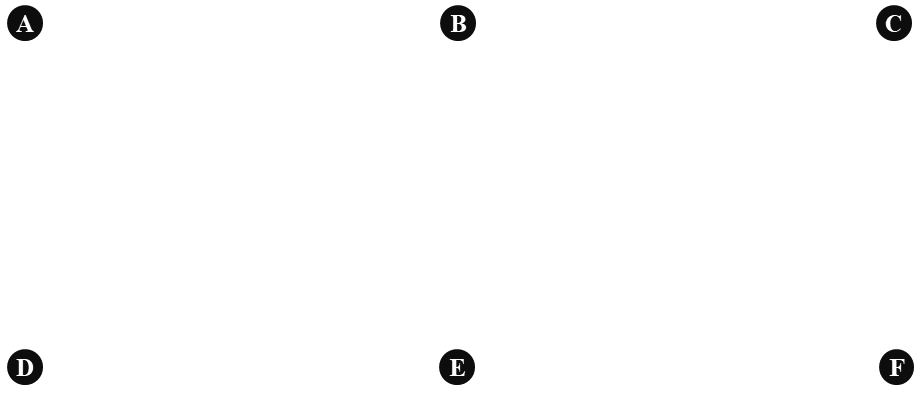

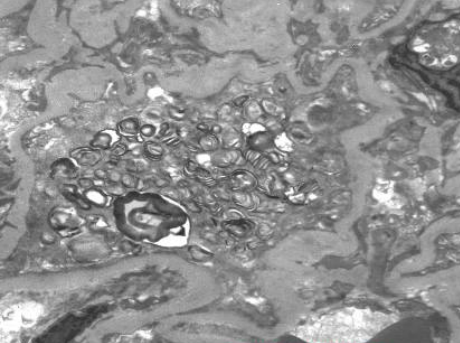

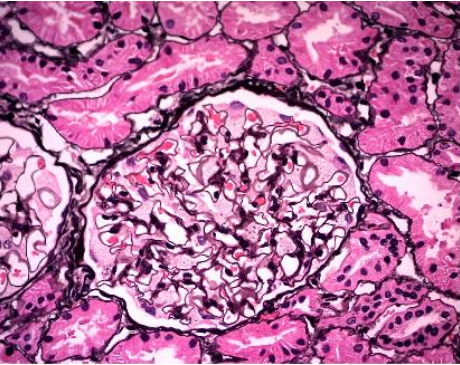

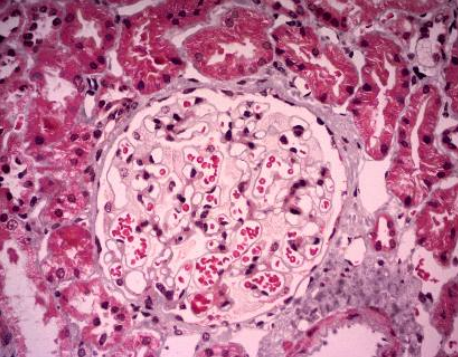

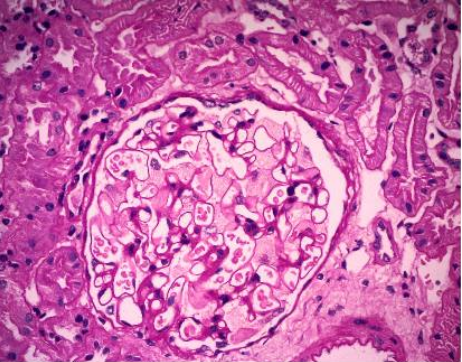

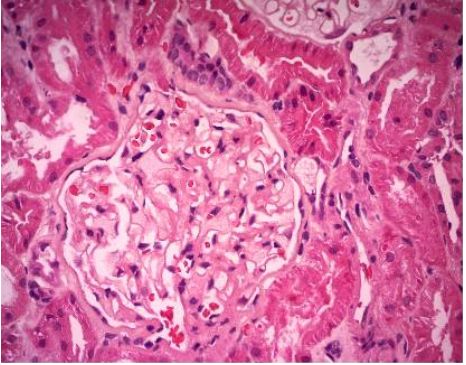

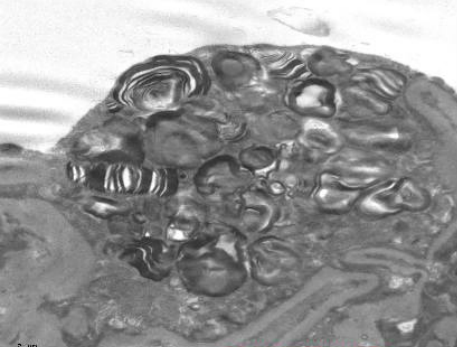


Supplemental Figure 1. Results of renal pathological. Light microscopy (A-D). (A) HE staining, × 400. (B) PAS staining, ×400. (C) PASM staining, ×400. (D) Masson staining, ×400. Electron microscopy (E-F). (E) × 200. (F) ×400.
